# NeuroBridge: a prototype platform for discovery of the long-tail neuroimaging data

**DOI:** 10.3389/fninf.2023.1215261

**Published:** 2023-08-31

**Authors:** Lei Wang, José Luis Ambite, Abhishek Appaji, Janine Bijsterbosch, Jerome Dockes, Rick Herrick, Alex Kogan, Howard Lander, Daniel Marcus, Stephen M. Moore, Jean-Baptiste Poline, Arcot Rajasekar, Satya S. Sahoo, Matthew D. Turner, Xiaochen Wang, Yue Wang, Jessica A. Turner

**Affiliations:** ^1^Psychiatry and Behavioral Health Department, The Ohio State University Wexner Medical Center, Columbus, OH, United States; ^2^Information Sciences Institute and Computer Science, University of Southern California, Los Angeles, CA, United States; ^3^Department of Medical Electronics Engineering, BMS College of Engineering, Bangalore, India; ^4^Department of Radiology, Washington University in St. Louis, St. Louis, MO, United States; ^5^Department of Neurology and Neurosurgery, McGill University, Montreal, QC, Canada; ^6^Renaissance Computing Institute, Chapel Hill, NC, United States; ^7^School of Information and Library Science, University of North Carolina at Chapel Hill, Chapel Hill, NC, United States; ^8^Department of Population and Quantitative Health Sciences, Case Western Reserve University, Cleveland, OH, United States; ^9^College of Information Sciences and Technology, Pennsylvania State University, State College, PA, United States

**Keywords:** addiction, schizophrenia, experimental design, MRI, metadata, ontology, text-mining

## Abstract

**Introduction:**

Open science initiatives have enabled sharing of large amounts of already collected data. However, significant gaps remain regarding how to find appropriate data, including underutilized data that exist in the long tail of science. We demonstrate the NeuroBridge prototype and its ability to search PubMed Central full-text papers for information relevant to neuroimaging data collected from schizophrenia and addiction studies.

**Methods:**

The NeuroBridge architecture contained the following components: (1) Extensible ontology for modeling study metadata: subject population, imaging techniques, and relevant behavioral, cognitive, or clinical data. Details are described in the companion paper in this special issue; (2) A natural-language based document processor that leveraged pre-trained deep-learning models on a small-sample document corpus to establish efficient representations for each article as a collection of machine-recognized ontological terms; (3) Integrated search using ontology-driven similarity to query PubMed Central and NeuroQuery, which provides fMRI activation maps along with PubMed source articles.

**Results:**

The NeuroBridge prototype contains a corpus of 356 papers from 2018 to 2021 describing schizophrenia and addiction neuroimaging studies, of which 186 were annotated with the NeuroBridge ontology. The search portal on the NeuroBridge website https://neurobridges.org/ provides an interactive Query Builder, where the user builds queries by selecting NeuroBridge ontology terms to preserve the ontology tree structure. For each return entry, links to the PubMed abstract as well as to the PMC full-text article, if available, are presented. For each of the returned articles, we provide a list of clinical assessments described in the Section “Methods” of the article. Articles returned from NeuroQuery based on the same search are also presented.

**Conclusion:**

The NeuroBridge prototype combines ontology-based search with natural-language text-mining approaches to demonstrate that papers relevant to a user’s research question can be identified. The NeuroBridge prototype takes a first step toward identifying potential neuroimaging data described in full-text papers. Toward the overall goal of discovering “enough data of the right kind,” ongoing work includes validating the document processor with a larger corpus, extending the ontology to include detailed imaging data, and extracting information regarding data availability from the returned publications and incorporating XNAT-based neuroimaging databases to enhance data accessibility.

## Introduction

The unprecedented data revolution has generated an enormous amount of data, including biomedical imaging datasets. In 2022, the NIH funded over 7,000 neuroimaging-related projects, encompassing virtually every institute (National Institutes of Health, National Institutes of Health). Over 6,000 currently open clinical trials rely on imaging as a primary endpoint or other key dependency^1^. Much of the present efforts on reproducibility science are focused on annotation, processing, and to some extent analysis. The new NIH Data Management and Sharing Policy (National Institutes of Health, 2023) is encouraging the sharing of data and has pointed to repositories for depositing data. However, how to find data, and more importantly, how to find sufficient data that is appropriate to answering a specific research question, is currently left to the individual researcher to navigate. The facilitation of finding sufficient data of the right kind is a critical gap.

Currently, much of the data is not yet “findable.” While organized, big neuroimaging data is being shared through mechanisms such as searchable archives (see an example list of the many different neuroimaging databases that are sharing data) (Eickhoff et al., 2016), and data are being reported and deposited with recently established resources such as data journals (Walters, 2020) and EuropePMC^2^, an even larger number of smaller-sized datasets have been collected in day-to-day research by individual laboratories and reported in peer-reviewed publications: approximately 9,000 full text papers are available at *Frontiers in Psychology* and *Frontiers in Neuroscience* alone, and Neurosynth.org contains 10,000 fMRI papers. Many of these datasets are utilized once and never shared. These underutilized “gray data” along with the rest of the data that remain in the unpublished “darkness” form the “long tail of data” (Wallis et al., 2013; Ferguson et al., 2014). Finding, accessing, and reusing these data could greatly enhance their value and lead to improved reproducibility science.

Searching the scientific literature for data is a labor intensive endeavor. While researchers can search for papers on platforms such as PubMed Central (PMC) and Google Scholar, culling through the returned articles to identify which ones may contain relevant study populations and whether they include references to datasets is time consuming. One coauthor’s Ph.D student wished to assess the reliability of automated tracing of the amygdala, and whether manual-vs-automated differences might account for disagreements in the literature. Through obtaining data directly from authors, she was able to definitively demonstrate that amygdala volumes were not a sensitive measure in the population she was researching, and that differences in tracing methodology did not account for the literature disagreements (Jayakar, 2017; Jayakar et al., 2018, 2020). However, this process took 18 months! A more efficient process by which researchers can find relevant data in the literature is needed.

To improve search efficiency, a large body of work has been done to annotate the research literature (Fox et al., 2005; Turner and Laird, 2012). PubMed, for example, tags papers with the Medical Subject Headings (MeSH) terms. In the neuroimaging community, the Neurosynth project has derived keywords and result tables from full text of functional MRI papers. The NeuroQuery platform developed a library of ∼7,500 keywords to label fMRI activation coordinates in full text papers on psychiatric studies (Dockes et al., 2020). Many scientific domains, including neuroscience, extensively adopt ontologies to describe observations and organize knowledge (Moreau et al., 2008; Widom, 2008; Sahoo et al., 2019). Using these ontologies to annotate textual descriptions of datasets is therefore a key step toward effective data discovery and selection. Natural language processing (NLP) and machine learning approaches have the potential to automate this process. For example, the Brainmap Tracker used the Cognitive Paradigm Ontology to guide text-mining for tagging papers (Laird et al., 2005; Turner and Laird, 2012; Turner et al., 2013; Chakrabarti et al., 2014). Traditional machine learning algorithms often require training on a large number of annotated examples, where unstructured texts are manually annotated using a complex ontology. This is a labor-intensive process that requires highly specialized domain expertise. We have previously developed a deep-learning classification algorithm that obtained high accuracy without assuming large-scale training data (Wang et al., 2022), by exploiting pre-training deep neural language models on rich semantic knowledge in the ontology.

In this context, we launched the NeuroBridge project to facilitate the discovery and reuse of neuroimaging data described in peer-reviewed publications and searchable databases. It is important to note that while there are efforts on modeling provenance metadata during the design and implementation of studies prior to publication (Keator et al., 2013; Gorgolewski et al., 2016; Kennedy et al., 2019), the NeuroBridge is focused on completed studies that are described in papers.

The NeuroBridge project supports the FAIR data principles (Wilkinson et al., 2016) for improving findability, accessibility, interoperability and reusability of scientific data in the following ways. *Findability*: FAIR recommends that metadata and data should be easy to find. NeuroBridge enhances the findability of data through clinical ontology-based indexing for finding presence of data usage in publications. *Accessibility*: FAIR recommends that a user be given information on how data can be accessed once found. In NeuroBridge we provide the data availability statement and author contact information that we extract automatically from publication metadata. *Interoperability*: FAIR recommends common vocabulary and use of formal, accessible, shared, and broadly applicable language for representation of data and metadata. NeuroBridge provides mappings between metadata terms used by data providers and published studies to metadata schemas that conform to standard terms or ontology. *Reusability*: FAIR recommends data be richly described with a plurality of accurate and relevant metadata attributes. NeuroBridge provides metadata schemas that are annotated with common vocabulary and ontology. We have made all of our relevant data and tools freely available^3^ ^,4^ to encourage the neuroscience community to produce data that can be legally and efficiently utilized by third party investigators.

Our long-term goal is to bridge the research question with data and scientific workflow, thereby significantly speeding up the cycle of hypothesis-based research. In the companion paper in this special issue, we describe the NeuroBridge ontology (Sahoo et al., 2023). In this paper, we report the NeuroBridge prototype platform that focused on neuroimaging studies of schizophrenia and addiction disorders as application domains. To extract metadata about study design and data collection from full-text papers, we leveraged a number of previous efforts on ontology development and machine-learning based natural-language processing.

## The NeuroBridge prototype architecture

**FIGURE 1:**
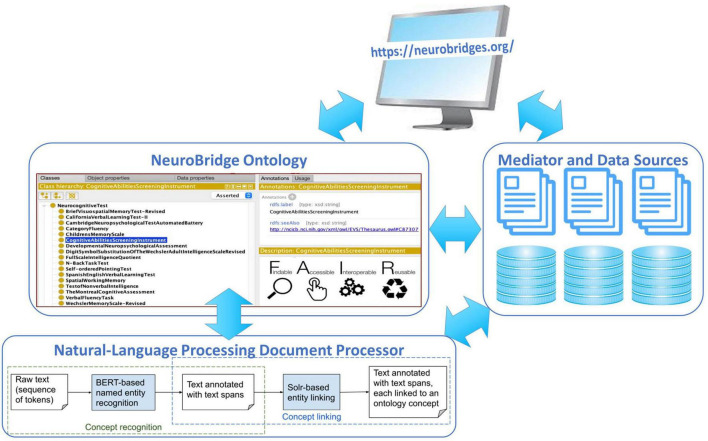
The NeuroBridge prototype architecture includes an extensible NeuroBridge ontology along with a set of full-text peer-reviewed papers annotated with the ontology, a natural-language processing document processor deep neural network model, and a user-friendly interface that contained an interactive query builder and integrated search across disparate data sources completed the prototype architecture.

The design of the NeuroBridge architecture (Figure 1) was guided by our overall goal to find enough data of relevance to the user, and by the principle of identifying relevance by metadata that is harmonized by a common ontology. Within this principle, we first created an extensible NeuroBridge Ontology that was interoperable with other domain-specific terminological systems such as the Systematized Nomenclature of Medicine Clinical Terms (SNOMED CT), the Neuroimaging Data Model (NIDM) ontology (Maumet et al., 2016), and the RadLex ontology developed by the Radiological Society of North America. This ontology was then used to annotate a set of full-text peer-reviewed papers, which was then used to train a natural-language document processor to develop a deep neural network model to represent each paper with the ontological concepts. Finally, a user-friendly interface that contained an interactive query builder and integrated search across disparate data sources completed the prototype architecture.

We first established a document corpus of PMC papers to develop the NeuroBridge ontology and train our deep neural network document processor. The corpus contained 356 full-text articles from 2017 to 2020, available from the National Library of Medicine (NLM) BioC collection, reporting empirical studies of schizophrenia and substance-related disorders that have collected neuroimaging data on human subjects, excluding meta-analysis and review papers. The NLM BioC collection (Comeau et al., 2019) is a simple format designed for straightforward text processing, text mining and information retrieval research, e.g., using plain text or JSON. Details of queries performed on PMC are shown in Table 1. Of the 356 articles, 186 were used to annotate with the NeuroBridge ontology and train our deep neural network document processor, described below.

**TABLE 1:** The prototype document corpus.

PMC search	Schizophrenia	Substance-related disorder
Search string	[“functional neuroimaging” (mh)] [“schizophrenia” (mh)] NOT [meta-analysis(pt) OR review(pt)] NOT [meta-analysis(ti) or review(ti)]	[“functional neuroimaging” (mh)] [“substance-related disorders” (mh)] NOT [meta-analysis(pt) or review(pt)] NOT [meta-analysis(ti) or review(ti)]
Additional PMC filters applied to both searches	Free full text; Time In the last 5 years; Subjects: Humans; language: English	
# of returns on PMC	335	200
# of articles retrieved from BioC	196	162
# of articles used in document collection	196 + 162–2 = 356 (two articles are common between the above two sets)	

## The NeuroBridge ontology

Full details of the ontology and its development process are described in the companion paper in this special issue (Sahoo et al., 2023). The NeuroBridge ontology was developed in the metadata framework called the S3 model that classified provenance metadata related to research studies into the categories of *study instrument*, *study data*, and *study method* (Sahoo et al., 2019), which extended the World Wide Web Consortium (W3C) PROV specification to represent provenance metadata for the biomedical domain. The NeuroBridge ontology was developed to be interoperable in annotating the neuroimaging literature and extensible to model additional study metadata such as subject recruitment and data collection methods. It incorporated our previous work on terminologies for data sharing in schizophrenia (Wang et al., 2016), and extended it to include metadata terms from the ENIGMA Addiction Project (Cao et al., 2021). It systematically and comprehensively modeled metadata information that described neuroscience experiments such as the number of participants in a diagnostic group, the type of experiment data collected (neuroimaging, neurophysiology etc.), and the clinical and cognitive assessment instruments.

The NeuroBridge ontology model included terms for neuroimaging data types for T1-weighted, task-based or resting-state functional imaging, a variety of clinical diagnoses such as neurodevelopmental disorder, mental disorders, and cognitive disorder. It also included various clinical and cognitive assessment instruments such as substance use scales, psychopathology scales, neurocognitive scales and mental health diagnosis scales. The ontology was integrated into the natural language processing pipeline and the NeuroBridge query interface, both described below, to allow use of metadata terms in composing user query expressions and identify relevant study articles.

The NeuroBridge ontology currently consists of 640 classes together with 49 properties that link the ontology classes. Using the ontology, we annotated 186 papers from our document corpus on the participant types, scanning, clinical and cognitive assessments. See the companion paper in this special issue for a more thorough presentation of the ontology and annotations (Sahoo et al., 2023), including the class hierarchy representing various diagnoses and assessment scales. The latest version of the NeuroBridge ontology is available on GitHub (NeuroBridge) (see text footnote 4) and will be made available on BioPortal soon.

## Ontology-based natural language document processor

The goal of the document processor was to extract from full-text articles in our corpus any relevant metadata information regarding study design and data collection as modeled by the NeuroBridge ontology. A key element of the design was to represent each full-text article in the corpus as a collection of the ontological concepts, instead of the original representation as a sequence of words in the full text. This eliminated the need to generate synonyms, hypernyms and hyponyms that are common in text-based search platforms. For the prototype reported here, the sample size of our corpus of annotated full-text papers was small relative to the number of ontological concepts (186 vs. 640, respectively, see above). This small sample size did not lend itself to an end-to-end deep-learning model that would simultaneously tag and classify text spans into the ontology terms. Our prior research on low-resource named entity recognition showed that when the training set was small and entity tokens were sparse, fine-tuning a pre-trained large language model had a consistent performance advantage over training simpler models such as conditional random fields or bi-directional long short-term memory (Wang and Wang, 2022). This led to the development of a two-stage machine-learning model, described in detail in Wang et al. (2022) and briefly outlined here.

Stage 1 of the model was concept recognition, where text spans in the full text that may mention any ontological concept term were tagged. This was formulated as a binary sequence tagging task to determine whether a text span should be recognized as *any* concept or not, regardless which concept it is linked to. We employed the Bidirectional Encoder Representations from Transformers (BERT) with a conditional random fields (CRF) output layer as the binary sequence tagging model. BERT is a deep neural network model for natural language (Devlin et al., 2019) that learns from a corpus of documents to obtain the contextual representation of a word using information from all other words in a sentence. This makes BERT especially powerful in fine-grained natural language processing tasks (both at a sentence and at the word level) where nuanced syntactic and semantic understanding is critical.

Then in Stage 2, concept linking, the tagged texts were mapped to the most relevant concept in the ontology. For each concept, we constructed a “concept document” by concatenating its textual labels in the NeuroBridge ontology, its synonyms in the UMLS, and its associated text spans in the training data. We then calculated the textual similarity between the text span and the concept document by using Apache Solr to index all concept documents where a text span was treated as a free-text query and the BM25 relevance model (Amati, 2009) was used to rank concepts. The textual similarity provided a measure of relevance of a text span with respect to a concept, which was used to train and develop the model. In the case where Solr returned no result for a given text span, we used fuzzy string matching (i.e., Jaccard similarity of two sets of letter trigrams) between the text span and a concept as a fallback strategy to rank the relevance to the concepts.

For each of the articles in our corpus (except those used for training), we applied the trained two-stage document processor on the Sections “Abstract” and “Methods” to create a representation as a collection of machine-recognized ontological concepts. During queries performed in the NeuroBridge search portal (described below), these representations would be used to match against the query criteria.

## Interactive search portal and integrated query across disparate sources

### Overview

When the user comes to the NeuroBridge search portal website (see text footnote 1), a typical workflow begins in the query builder interface with the construction of a query by the user selecting a series of NeuroBridge ontology terms as search keywords. The query is then passed to the backend to search across the different data sources. Returns from each data source are then listed for further exploration by the user.

### Query construction

**FIGURE 2:**
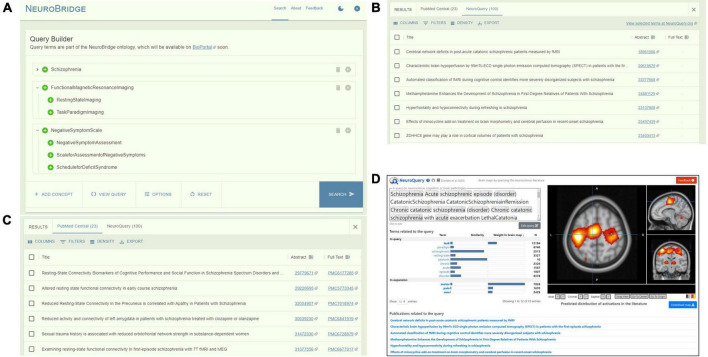
The NeuroBridge portal of an example query of “Schizophrenia” AND “FunctionalMagneticResonanceImaging” AND “NegativeSymptomScale.” **(A)** Query builder interface showing query construction on the ontology concepts that automatically included all descendants. **(B)** Returns from PubMed Central with links to full-text articles. **(C)** Returns from NeuroQuery with links to PubMed abstracts. **(D)** User is directed to the NeuroQuery portal for direct interaction.

In the Query Builder window, the user types in parts of the keyword that they want to query on, and the Query Builder will present a list of suggested ontology concept terms based on the spelling of the partial keyword. By default, all descendants of the selected ontology concept term will be included and the user can include and exclude individual descendants. The user can continue to add additional ontology concept terms to the query. An example query is shown in Figure 2A, constructed on the ontological concepts of “Schizo- phrenia,” “FunctionalMagneticResonanceImaging,” “Negative- SymptomScale,” with all the descendants of these terms automatically included into the query.

The portal front-end will form the final query by joining the terms together with Boolean logics of “AND” and “OR,” and represents it in a JSON format to preserve the ontology tree structure. A “View Query” option on the Query Builder portal allows the user to inspect the query syntax before submitting for execution. Upon user submission, the Boolean-represented query is then sent to the backend to be matched against the ontology representations of the full-text articles in the document corpus, as described above.

### Query across disparate sources

For the same query the user constructed, we have also implemented mediation strategies to search additional data sources. In the current NeuroBridge prototype, in addition to the PMC articles corpus, we have incorporated NeuroQuery (Dockes et al., 2020) as a second data source and are currently working on incorporating XNAT (Marcus et al., 2007a) data sources. NeuroQuery is a platform that provides fMRI activation maps along with PubMed source articles (Dockes et al., 2020). It has a native search interface for user-input free texts and returns which terms, PMC publications, and brain regions are related to the query. The matching within NeuroQuery is based on its library of ∼7,500 native terms and ∼13,000 PMC neuroimaging articles.

We directed the NeuroBridge search to NeuroQuery by employing ElasticSearch and SapBERT (2023)^5^ to semantically match terms in the NeuroBridge ontology to the native NeuroQuery terms so that terms being queried at NeuroBridge can be translated to NeuroQuery native terms. The translation process started by using SapBERT to create a floating-point vector of dimension 768 for each of NeuroQuery’s native terms. These vectors represented the position in SapBERT’s feature space of each of the terms. The vectors were then loaded into an ElasticSearch index that could be accessed by a Flask based API. To translate a NeuroBridge term to a NeuroQuery term, the API used SapBERT to create a corresponding vector for the NeuroBridge term. Then using the Cosine Similarity capability in ElasticSearch, the vector representing the NeuroBridge term was compared to the vector representing each of the NeuroQuery vectors to select the closest match. As an example, suppose the user has selected the NeuroBridge term “abstinent.” Searching NeuroQuery using its native API did not return any data. Searching the ElasticSearch index for the closest match to abstinent selected the NeuroQuery term “abstinence.” Using the NeuroQuery native API with this term returned several matches. The use of ElasticSearch and SapBERT enabled searching the NeuroQuery API using its native term set while still enabling the user to search using the NeuroBridge ontology.

### Return exploration

In the Results panel, returns of the query from each data source are presented to the user in its own tab. For returns from the PMC article corpus, the returns are sorted by relevance as computed above. Figure 2B shows that the query on the terms “Schizophrenia,” “Functional Magnetic Resonance Imaging,” “NegativeSymptomScale,” and all their descendants resulted in a return of 23 PMC articles from the NeuroBridge corpus. For each return entry, links to the PubMed abstract as well as to the PMC full-text article, if available, are presented.

The same query resulted in a return of 100 articles from NeuroQuery (Figure 2C) (note: NeuroQuery by default returns 100 articles ranked by relevance from their corpus of ∼13,000 articles). A link to the NeuroQuery portal is also provided for users who are interested in interacting directly with NeuroQuery (Figure 2D).

**FIGURE 3:**
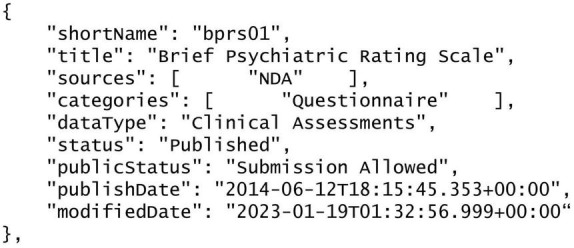
One of the more than 4,400 common assessment instruments from the National Institute of Mental Health Data Archive (NDA), the assessment “Brief Psychiatric Rating Scale,” in JSON format.

**FIGURE 4:**
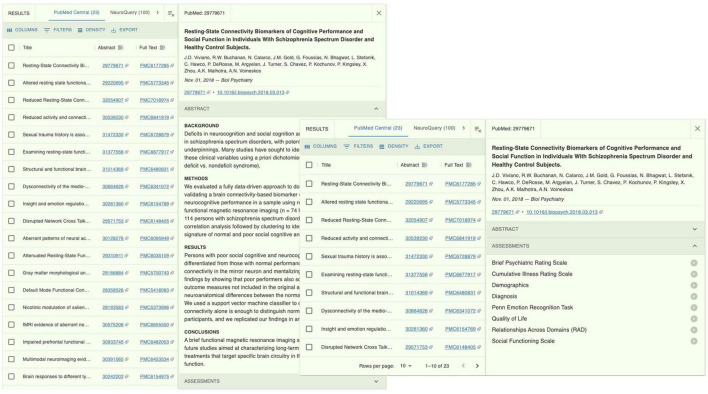
The NeuroBridge portal of returns on the example query in Figure 2, showing a list of clinical assessments described in the full-text article.

We experimented with additional capabilities on the returned articles for providing useful information to the user. One kind of useful information is the set of clinical, behavioral and cognitive assessments that a study may have used. We first extracted a list of >4,400 names of common assessment instruments from the National Institute of Mental Health Data Archive (NDA). NDA is an informatics platform that supports data sharing across all mental health and other research communities. The list of assessment instruments thus spans across all mental health conditions^6^. The extracted list was in JSON format, where each assessment has a unique “title” (e.g., “Brief Psychiatric Rating Scale”) and a unique “shortName” (e.g., “bprs01”). See Figure 3 for an example entry. We used the Apache Solr-based method we employed in the Document Processor (see previous section) to compute textual similarities between the assessment “title” and the texts in the Section “Methods” of the paper. Matched items were collated for each returned article and presented to the user. For example, for the returned article (PMCID PMC6177285) (Viviano et al., 2018), the assessments included “Brief Psychiatric Rating Scale,” “Cumulative Illness Rating Scale,” “Penn Emotion Recognition Task,” and “Social Functioning Scale” (Figure 4).

**FIGURE 5:**
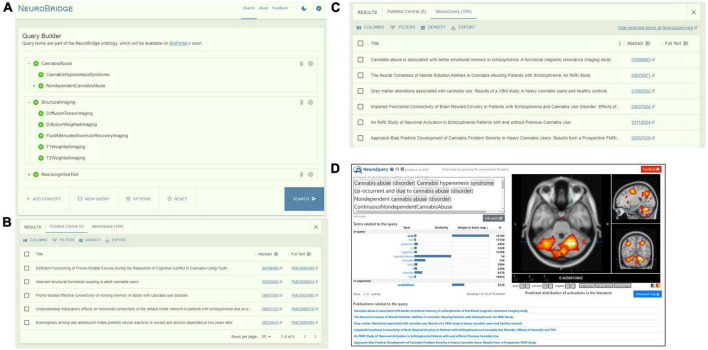
An example query of “CannabisAbuse” AND “StructuralImaging” AND “NeurocognitiveTest.” **(A)** Query builder interface showing query construction on the ontology concepts that automatically included all descendants. **(B)** Returns from PubMed Central with links to full-text articles. **(C)** Returns from NeuroQuery with links to PubMed abstracts. **(D)** User is directed to the NeuroQuery portal for direct interaction.

As another example, we built a query using concepts “CannabisAbuse,” “StructuralImaging,” “NeurocognitiveTest” and their descendants (Figure 5A). Figures 5B–D show the returned PMC articles from the NeuroBridge corpus and results from NeuroQuery.

### Power of ontology-based search

**FIGURE 6:**
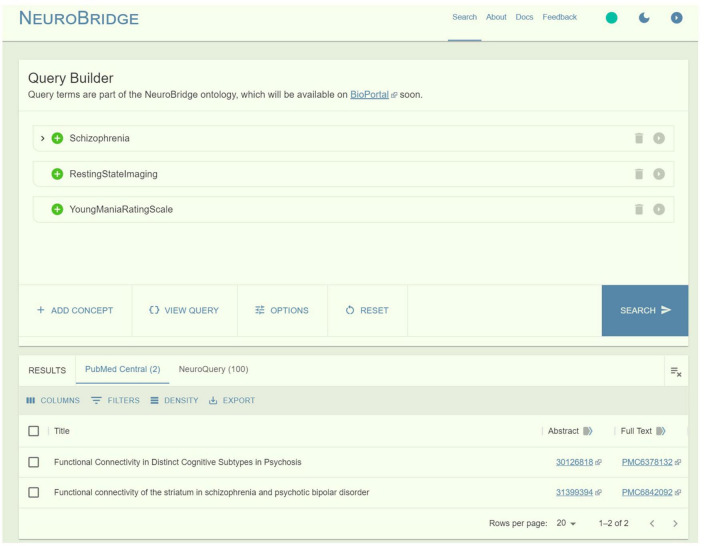
The power of ontology-based search, as demonstrated by a query of “Schizophrenia” AND “Resting-State Imaging” AND “Young Mania Rating Scale”: On NeuroBridge, two articles were returned.

**FIGURE 7:**
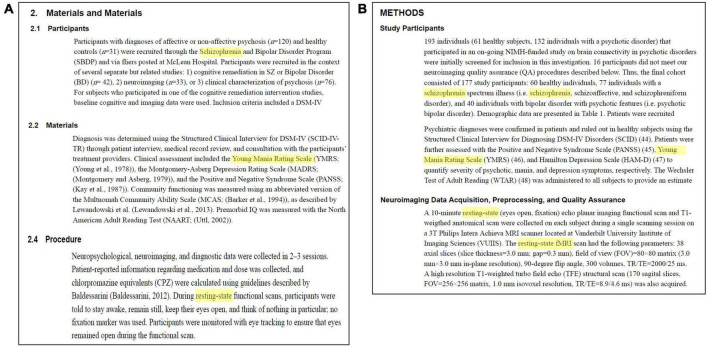
The power of ontology-based search, continued, as demonstrated by the query shown in Figure 6: Relevant text snippets in panel **(A)** Lewandowski et al. (2019) and **(B)** Karcher et al. (2019). In comparison, the direct search on the PMC portal failed to return any entries.

To demonstrate the power of ontology-based search, we compared query results of “Schizophrenia,” “Resting-State Imaging,” and “Young Mania Rating Scale” between NeuroBridge and a direct search on PMC. On the NeuroBridge search portal, two articles were returned: Lewandowski et al. (2019) “Functional Connectivity in Distinct Cognitive Subtypes in Psychosis” (PMC6378132) and Karcher et al. (2019) “Functional Connectivity of the Striatum in Schizophrenia and Psychotic Bipolar Disorder” (PMC6842092) (Figure 6). In Lewandowski et al. (2019), the Sections “Methods” included the terms “schizophrenia,” “Young Mania Rating Scale (YMRS),” and “resting-state functional scans” (Figure 7A). In Karcher et al. (2019), the Sections “Methods” included the terms “schizophrenia,” “Young Mania Rating Scale (YMRS),” and “resting-state fMRI” (Figure 7B). In comparison, the direct search on the PMC portal failed to return any entries. Additional synonyms such as “Resting-State fMRI” or “Resting-State functional” resulted in returns from the PMC. While the returns included the above articles, they also included many false positives. For example, the article by Gallucci et al. (2022) “Longer illness duration is associated with greater individual variability in functional brain activity in Schizophrenia, but not bipolar disorder” (PMC9723315) included the terms “schizophrenia” and “Young Mania Rating Scale (YMRS)” in the Section “Methods,” the study did not utilize resting-state fMRI - subjects performed the N-back fMRI only.

## Discussion

In this paper we describe the NeuroBridge: a project that takes a first step toward the discovery of gray neuroimaging data for reuse. The term “gray data” refers to data that has been gathered and used for analysis but is not publicly available. Reuse of these data is economic (i.e., compared with the large amount of funding required to collect new data) and can enhance reproducibility research (e.g., by facilitation of replication as well mega-analysis of aggregated data). Traditionally, finding data has been done mainly through professional networking and manually searching the literature^7^. However, much of the data mentioned in publications has not been shared yet through data links (such as DOI) or described in any searchable databases. Few resources currently exist that can help researchers find the right kind of data described in publications that are appropriate for their research questions.

Recent efforts have begun to facilitate these searches. For example, the field of life sciences requires papers to be deposited in domain repositories and uses DOIs to help to make data search easier than before. Data journals publish peer-reviewed documentation of data and provide repositories for the data (Walters, 2020). The EuropePMC platform (see text footnote 2) provides links to data related to publications, but only data that has a citation or has been uploaded to one of the known 40 biological databases. Despite progress on improving data sharing and data curation, a wide gap exists in finding such data of interest for a user. Many of the legacy articles do not have explicit data citations and there is no standard in which articles detail the data gathering and data analysis operations. Finding whether there are any references to data of interest in these articles is a time-consuming manual process. Currently, finding data described in publications involves manually searching the literature, identifying the returned articles that are closely related to a research topic, and checking if the datasets described in the articles are appropriate and if the articles include references to the datasets.

The NeuroBridge prototype platform described in this paper aims to ease the burden for the user and takes a first step toward the discovery of gray neuroimaging data for reuse. NeuroBridge is powered by a machine learning system that is trained to identify clinical neuroscience metadata terms, including diagnosis, MRI scan types, and clinical assessments in a subset of articles that are accessible through PubMed Central. The current prototype is trained with an ontology in the domains of schizophrenia and substance-use disorders along with the clinical terms to facilitate discovery of relevant neuroimaging data described in peer-reviewed full-text journal papers. In the prototype platform, the user can perform a keyword-based search related to their research question, examine the returned papers for types of clinical assessment data, and pursue data access either via the data availability information or author contacts, both of which are provided in the NeuroBridge search returns.

### Related work

The long-term goal of the NeuroBridge project is to provide researchers who are searching for neuroimaging data for a specific project (e.g., meta or mega analysis of a specific neuroimaging type in a specific clinical domain) with sufficient data of the right kind. Toward this goal, the NeuroBridge prototype reported here builds upon a number of previous and ongoing efforts on building ontologies of study design and text mining.

To discover studies that may contain relevant data for the user, we rely on the provenance metadata that can model study population, design and data collection. Extensive research on provenance metadata collection, storage, and querying has led to the emergence of relational databases, scientific workflow systems, sensor networks, and Semantic Web applications (Moreau et al., 2008; Widom, 2008; Sahoo et al., 2019). The biomedical research community has developed ontologies to model metadata information associated with clinical trials such as eligibility criteria (Tu et al., 2011; Sim et al., 2014). In the neuroscience research community, several recent initiatives have made significant progress toward identifying metadata information that can be used to describe the context of studies. These initiatives include the Neuroimaging Data Model (NIDM) (Keator et al., 2013), Reproducible Neuroimaging Computation (ReproNim) (Kennedy et al., 2019), and Brain Imaging Data Structure (BIDS) (Gorgolewski et al., 2016). These efforts, however, are focused on prospective studies on modeling provenance metadata prior to publication, while the goal of the NeuroBridge modeling approach is to help locate completed studies that are described in papers.

Many semantic search systems index unstructured content (usually text) using concepts or terms in a target ontology and allow users to query the content using these terms. The most prominent system is PubMed, which indexes the biomedical literature using terms in Medical Subject Headings (MeSH) and allows users to use MeSH terms in their search queries. The MeSH terms are currently automatically assigned to each PubMed paper using the MTI system^8^ ^,9^, with a selected subset of papers reviewed by human indexers for quality control. Another system is LitCovid, which annotates and searches COVID-19-related research articles with medical terms such as genes, diseases, and chemical names (Chen et al., 2021). Other search engine prototypes such as SemEHR (Wu et al., 2018) and Thalia (Soto et al., 2019) assign terms in the Unified Medical Language System (UMLS) to documents and use these terms as search facets. The radiology image search engine prototype GoldMiner (Kahn and Thao, 2007) assigns terms in Systematized Nomenclature of Medicine Clinical Terms (SNOMED CT) and MeSH terms to image documents to facilitate image search. A key advantage of these systems compared to keyword-based search engines is that they allow users to directly use ontological concepts to express specific information needs that are otherwise challenging to precisely express through keywords.

A significant amount of research efforts has been dedicated to extracting semantic concepts from unstructured text. The problem is referred to as semantic indexing when the extracted concepts are used to represent texts in an information retrieval system (Reinanda et al., 2020). The problem is usually formulated as a natural language processing task, such as named entity recognition (Li et al., 2022), entity linking (Shen et al., 2015), or multi-label text classification (Mao and Lu, 2017). To solve these tasks, machine learning techniques are often employed. A machine learning system learns from a set of articles with human-assigned terms as training examples and generates a model that generalizes the term assignment procedure from the training articles to new unlabeled articles. Neural language models such as BERT (Devlin et al., 2019) can often deliver state-of-the-art performance on these tasks. These models learn rich prior knowledge from large-scale unlabeled text in their pre-training stage, which makes them easily adaptable to specific tasks by fine-tuning on a relatively small training dataset. A recent platform Elicit^10^ uses Generative Pre-trained Transformer (GPT) to find papers related to a research question based on semantic similarity. For NeuroBridge, the ability to quickly learn from a small training dataset is important since it is expensive and time-consuming to curate even a moderate amount of biomedical research articles with concepts in a complex ontology. We have previously developed a deep-learning classification algorithm without large-scale training data (Wang et al., 2022). This was achieved by exploiting BERT that had been pre-trained on large unannotated text corpus and further fine-tuning it on annotated data that encoded rich semantic knowledge in the ontology. The technique could generalize to a wide range of biomedical text mining scenarios where the target ontological structure is complex but constructing large training data sets is too expensive and time-consuming.

Currently, a researcher can pursue the following ways to find data for their research question: utilize their professional network and institutional resources such as data search engines available at institutional libraries (e.g., University of Bath, 2023), search known data repositories such as ones listed in Eickhoff et al. (2016), search indices of datasets such as DataCite’s Metadata Search^11^. The researcher can also search the literature. A number of journals in the field of biology, medicine and health sciences such as Scientific Data, Journal of Open Psychology Data, and Open Health Data are dedicated to the documentation and access of data created through research (Walters, 2020). While an increasing number of researchers are documenting their newly collected data in data journals, valuable, legacy data remain hidden in the literature. However, searches for data in the literature are performed by the researcher searching on literature databases such as PubMed Central, Open Science Framework then reading through each paper. There appears to be no current effort of systematically aiding this process. The abovementioned Elicit platform (see text footnote 10) offers advanced features such as extracting the number of participants and detailed study designs (e.g., case-control design, use of fMRI). To our knowledge, the NeuroBridge project is the first of its kind that is aimed at searching for relevant neuroimaging data described in peer-reviewed full-text papers.

## Conclusion and future work

The NeuroBridge prototype we presented here uses an ontology-based approach to facilitate the search for relevant peer-reviewed journal papers. While limitations exist, such as the small sample size of our training and testing corpus, it nevertheless takes an important first step toward identifying potential neuroimaging data described in full-text papers that are relevant to a particular user’s research interests. Work is ongoing to validate the document processor with a larger corpus, extend the ontology to include detailed imaging data, extract information regarding data availability from the returned publications to enhance data accessibility (FAIR), and measure semantic distances between studies based on assessment information to help identify relevance of studies to the user (Lander et al., 2019). Future work also involves extending the ontology and document corpus to include additional clinical domains (e.g., psychosis spectrum, dementia). These extensions will require similarly significant human effort including manually labeling a training set of papers with the ontology terms and careful review and curation of this work. See the companion paper in this issue for more detail of the labeling methods (Sahoo et al., 2023). As the system grows, the current iteration of the system supports this human labeling process by providing draft labels, and the entity-recognition, entity-linking, 2-stage natural language model will be retrained to complete the extension.

There is an increasing availability of multi-modal datasets in neuroscience research, especially as a result of the National Institutes of Health (NIH) Brain Research Through Advancing Innovative Neurotechnologies (BRAIN) initiative. NIH has developed large-scale data repositories such as the National Institute of Mental Health (NIMH) Data Archive (NDA) that contains datasets from structural and functional MRI, clinical phenotypes, and genomics. Eickhoff et al. (2016) described >40 neuroimaging data repositories across multiple clinical domains. A need exists to develop a metadata-based search and discovery platform on similar search criteria. Work is ongoing at the NeuroBridge project to incorporate XNAT-based (Marcus et al., 2007a) neuroimaging databases into our search. XNAT is a web-based software platform designed to facilitate common management and productivity tasks for imaging and associated data. It has been broadly adopted across domains of neuroscience, cardiology, cancer, and ophthalmology, supporting a wide range of many high impact data sharing initiatives, including OASIS (Marcus et al., 2007b,^2010^), Dementia Platform UK, Human Connectome Project (Hodge et al., 2016), UK Biobank (Miller et al., 2016), NITRC Image Repository (Kennedy et al., 2015), and SchizConnect (Wang et al., 2016). These resources offer comprehensive data from deep phenotyping of subjects, including multiple imaging modalities and clinical, cognitive, behavior, and genomic data. As the number of datasets rapidly grows, often the problem is not finding datasets, but selecting enough data of the right kind from a large corpus of possible datasets.

Our long-term goal is to discover “enough data of the right kind” by providing a user-friendly portal for automatically searching multiple types of sources and identifying relevant datasets. We envision a scenario where a graduate student or a postdoctoral fellow from a small institution can use NeuroBridge to discover data for testing specific hypotheses. For example, she may have read an interesting paper on how changes in brain networks are modulated by cognitive demand but the effects are different by sex. She would like to design a study to test the hypothesis or replicate the paper’s findings. However, her lab does not have the resources or budget for MR scanning or subject recruitment, and she can find only a very limited amount of data fitting her research needs in public databases. The student would then need to search through the literature to find data that are similar to the original study. It would take her an inordinate amount of time to comb through the details described in papers and decide whether they have the required data.

Additional future work of the NeuroBridge project includes: extracting detailed information on details of the study such as study design, sample demographic information as well as author contacts and data availability described in research papers, and identifying the location and links to such data if shared (through collaboration with platforms such as Brainlife (Avesani et al., 2019)^12^ where shared data are associated with publications. In the not too distant future, researchers like this student would interact with the NeuroBridges.org and its APIs, describe a study, craft their hypothesis, and in a few steps discover how many studies and datasets contain subjects and data that can be used to answer their research question. Our platform will become a key component of the data sharing ecosystem that provides researchers with sustainable means of aggregating data–from discovery, to access and harmonization – that are directly relevant to their hypothesis, and compute on the data to test their hypotheses. It will enable more small-market scientists to do large-scale research and thus increase the findability, accessibility, and reusability of scientific data to a greater number of researchers. We believe our approach can become the prototype in other domains for bridging from the research question, to data, to scientific workflow, thereby significantly speeding up the cycle of hypothesis-based research.

## Data availability statement

Publicly available datasets were analyzed in this study. This data can be found here: the NeuroBridge ontology: https://github.com/NeuroBridge/neuro-ontologies/tree/main/neurobridge.

## Author contributions

LW, JA, HL, AR, and JT contributed to the conception and design of the study. SS, AA, AK, MT, XW, YW, and JT developed the document corpus and the ontology and its annotations. XW and YW developed the natural-language based document processor. JD, HL, and J-BP contributed to the connection with NeuroQuery. JA, JB, RH, DM, and SM contributed to data mediation. HL coordinated the development of the search portal. LW wrote the first draft of the manuscript. XW, YW, HL, AR, MT, and JT wrote sections of the manuscript. All authors contributed to manuscript revision and read and approved the submitted version.

## Footnotes

1 ClinicalTrials.gov: https://www.clinicaltrials.gov/

2 Europe PubMed Central: https://europepmc.org/

3 NeuroBridge (Website): https://github.com/NeuroBridge/NeuroBridge1.0

4 NeuroBridge (Ontology): https://github.com/NeuroBridge/neuro-ontologies/tree/main/neurobridge

5 SapBERT, 2023: https://github.com/cambridgeltl/sapbert

6 Nimh Data Archive [NDA]: https://nda.nih.gov/general-query.html?q=query=data-structure%20%7Eand%7E%20dataTypes=Clinical%20Assessments%20%7Eand%7E%20orderBy=shortName%20%7Eand%7E%20orderDirection=Ascending%20%7Eand%7E%20resultsView=table-view

7 Wageningen University & Research: https://www.wur.nl/en/Library/Researchers/Finding-sources/Finding-research-data.htm

8 National Library of Medicine (NLM Medical Text Indexer): https://lhncbc.nlm.nih.gov/ii/tools/MTI.html

9 National Library of Medicine (Automated Indexing FAQs): https://support.nlm.nih.gov/knowledgebase/article/KA-05326/en-us

10 Elicit: https://elicit.org/

11 DataCite: https://commons.datacite.org/

12
https://brainlife.io/

## References

[B1] Amati G. (2009). “BM25,” in *Encyclopedia of database systems*, eds Liu L. Özsu M. T. (Boston, MA: Springer).

[B2] Avesani P. McPherson B. Hayashi S. Caiafa C. F. Henschel R. Garyfallidis E. (2019). The open diffusion data derivatives, brain data upcycling via integrated publishing of derivatives and reproducible open cloud services. *Sci. Data* 6:69. 10.1038/s41597-019-0073-y 31123325 PMC6533280

[B3] Cao Z. Ottino-Gonzalez J. Cupertino R. B. Schwab N. Hoke C. Catherine O. (2021). Mapping cortical and subcortical asymmetries in substance dependence: Findings from the ENIGMA Addiction Working Group. *Addict. Biol.* 26:e13010. 10.1111/adb.13010 33508888 PMC8317852

[B4] Chakrabarti C. Jones T. B. Luger G. F. Xu J. F. Turner M. D. Laird A. R. (2014). Statistical algorithms for ontology-based annotation of scientific literature. *J. Biomed. Semant.* 5:S2. 10.1186/2041-1480-5-S1-S2 PMC4108869 25093071

[B5] Chen Q. Allot A. Lu Z. (2021). LitCovid: An open database of COVID-19 literature. *Nucleic Acids Res.* 49 D1534–D1540. 33166392 10.1093/nar/gkaa952 PMC7778958

[B6] Comeau D. C. Wei C. H. Islamaj D. Lu Z. (2019). PMC text mining subset in BioC: About three million full-text articles and growing. *Bioinformatics* 35 3533–3535. 10.1093/bioinformatics/btz070 30715220 PMC6748740

[B7] Devlin J. Chang M. W. Lee K. Toutanova K. (2019). Bert: Pre-training of deep bidirectional transformers for language understanding. *arXiv* [Preprint]. 10.48550/arXiv.1810.04805

[B8] Dockes J. Poldrack R. A. Primet R. Gozukan H. Yarkoni T. Suchanek F. (2020). NeuroQuery, comprehensive meta-analysis of human brain mapping. *Elife* 9:e53385. 10.7554/eLife.53385 32129761 PMC7164961

[B9] Eickhoff S. Nichols T. E. Van Horn J. D. Turner J. A. (2016). Sharing the wealth: Neuroimaging data repositories. *Neuroimage* 124 1065–1068. 10.1016/j.neuroimage.2015.10.079 26574120 PMC5463741

[B10] Ferguson A. R. Nielson J. L. Cragin M. H. Bandrowski A. E. Martone M. E. (2014). Big data from small data: Data-sharing in the ‘long tail’ of neuroscience. *Nat. Neurosci.* 17 1442–1447. 10.1038/nn.3838 25349910 PMC4728080

[B11] Fox P. T. Laird A. R. Fox S. P. Fox P. M. Uecker A. M. Crank M. (2005). BrainMap taxonomy of experimental design: Description and evaluation. *Hum. Brain Mapp.* 25 185–198. 10.1002/hbm.20141 15846810 PMC6871758

[B12] Gallucci J. Pomarol-Clotet E. Voineskos A. N. Guerrero-Pedraza A. Alonso-Lana S. Vieta E. (2022). Longer illness duration is associated with greater individual variability in functional brain activity in Schizophrenia, but not bipolar disorder. *Neuroimage Clin.* 36:103269. 10.1016/j.nicl.2022.103269 36451371 PMC9723315

[B13] Gorgolewski K. J. Auer T. Calhoun V. D. Craddock R. C. Das S. Duff E. P. (2016). The brain imaging data structure, a format for organizing and describing outputs of neuroimaging experiments. *Sci. Data* 3:160044. 10.1038/sdata.2016.44 27326542 PMC4978148

[B14] Hodge M. R. Horton W. Brown T. Herrick R. Olsen T. Hileman M. E. (2016). ConnectomeDB–Sharing human brain connectivity data. *Neuroimage* 124 1102–1107. 10.1016/j.neuroimage.2015.04.046 25934470 PMC4626437

[B15] Jayakar R. (2017). *Amygdala volume and social anxiety symptom severity: A mutli-method Study, psychology.* Atlanta, GA: Georgia State University.

[B16] Jayakar R. Tone E. B. Crosson B. Turner J. A. Anderson P. L. Phan K. L. (2020). Amygdala volume and social anxiety symptom severity: Does segmentation technique matter? *Psychiatry Res. Neuroimaging* 295:111006. 10.1016/j.pscychresns.2019.111006 PMC6982531 31760338

[B17] Jayakar R. Tone E. B. Crosson B. A. Turner J. A. Anderson P. L. Phan K. L. (2018). “Association between amygdala volume and social anxiety symptom severity: A multi-method study,” in *46th Annual Meeting of the International Neuropsychological Society*, (Washington, DC).

[B18] Kahn C. E. Jr. Thao C. (2007). GoldMiner: A radiology image search engine. *AJR* 188 1475–1478. 17515364 10.2214/AJR.06.1740

[B19] Karcher N. R. Rogers B. P. Woodward N. D. (2019). Functional connectivity of the striatum in schizophrenia and psychotic bipolar disorder. *Biol. Psychiatry Cogn. Neurosci. Neuroimaging* 4 956–965. 31399394 10.1016/j.bpsc.2019.05.017 PMC6842092

[B20] Keator D. B. Helmer K. Steffener J. Turner J. A. Van Erp T. G. Gadde S. (2013). Towards structured sharing of raw and derived neuroimaging data across existing resources. *Neuroimage* 82 647–661. 10.1016/j.neuroimage.2013.05.094 23727024 PMC4028152

[B21] Kennedy D. N. Abraham S. A. Bates J. F. Crowley A. Ghosh S. Gillespie T. (2019). The repronim perspective on reproducible neuroimaging. *Front. Neuroinform* 13:1. 10.3389/fninf.2019.00001 30792636 PMC6374302

[B22] Kennedy D. N. Haselgrove C. Riehl J. Preuss N. Buccigrossi R. (2015). The three NITRCs: a guide to neuroimaging neuroinformatics resources. *Neuroinformatics* 13 383–386. 10.1007/s12021-015-9263-8 25700675 PMC4470758

[B23] Laird A. R. Lancaster J. L. Fox P. T. (2005). BrainMap: The social evolution of a human brain mapping database. *Neuroinformatics* 3 65–78. 10.1385/ni:3:1:065 15897617

[B24] Lander H. Alpert K. Rajasekar A. Turner J. Wang L. (2019). “Data Discovery for Case Studies: The DataBridge for Neuroscience Project,” in *Proceeding of the 13th International Multi-Conference on Society, Cybernetics and Informatics*, (Orlando, FL), 19–25.

[B25] Lewandowski K. E. McCarthy J. M. Ongur D. Norris L. A. Liu G. Z. Juelich R. J. (2019). Functional connectivity in distinct cognitive subtypes in psychosis. *Schizophr. Res.* 204 120–126. 30126818 10.1016/j.schres.2018.08.013 PMC6378132

[B26] Li J. Sun A. Han J. Li C. (2022). A survey on deep learning for named entity recognition. *IEEE Trans. Knowl. Data Eng.* 34 50–70.

[B27] Mao Y. Lu Z. (2017). MeSH Now: Automatic MeSH indexing at PubMed scale via learning to rank. *J. Biomed. Semant.* 8:15. 10.1186/s13326-017-0123-3 28412964 PMC5392968

[B28] Marcus D. S. Fotenos A. F. Csernansky J. G. Morris J. C. Buckner R. L. (2010). Open access series of imaging studies: Longitudinal MRI data in nondemented and demented older adults. *J. Cogn. Neurosci.* 22 2677–2684. 10.1162/jocn.2009.21407 19929323 PMC2895005

[B29] Marcus D. S. Olsen T. R. Ramaratnam M. Buckner R. L. (2007a). The Extensible Neuroimaging Archive Toolkit: An informatics platform for managing, exploring, and sharing neuroimaging data. *Neuroinformatics* 5 11–34. 10.1385/ni:5:1:11 17426351

[B30] Marcus D. S. Wang T. H. Parker J. Csernansky J. G. Morris J. C. Buckner R. L. (2007b). Open Access Series of Imaging Studies (OASIS): Cross-sectional MRI data in young, middle aged, nondemented, and demented older adults. *J. Cogn. Neurosci.* 19 1498–1507. 10.1162/jocn.2007.19.9.1498 17714011

[B31] Maumet C. Auer T. Bowring A. Chen G. Das S. Flandin G. (2016). Sharing brain mapping statistical results with the neuroimaging data model. *Sci. Data* 3:160102. 10.1038/sdata.2016.102 PMC5139675 27922621

[B32] Miller K. L. Alfaro-Almagro F. Bangerter N. K. Thomas D. L. Yacoub E. Xu J. (2016). Multimodal population brain imaging in the UK Biobank prospective epidemiological study. *Nat. Neurosci.* 19 1523–1536. 27643430 10.1038/nn.4393 PMC5086094

[B33] Moreau L. Ludascher B. Altintas I. Barga R. S. Bowers S. Callahan S. (2008). The provenance challenge. *Concurr. Comput. Pract. Exper.* 20 409–418.

[B34] National Institutes of Health. *NHI Reporter.* Vienna, VA: NHI.

[B35] National Institutes of Health (2023). *Data management and sharing policy.* Vienna, VA: NHI.

[B36] Reinanda R. Meij E. de Rijke M. (2020). Knowledge graphs: An information retrieval perspective. *Found. Trends Inform. Retrieval* 14 289–444.

[B37] Sahoo S. S. Turner M. D. Wang L. Ambite J. L. Appaji A. Rajasekar A. (2023). NeuroBridge ontology: Computable provenance metadata to give the long tail of neuroimaging data a FAIR chance for secondary use. *Front Neuroinform*. 17:1216443. 10.3389/fninf.2023.1216443 PMC10406126 37554248

[B38] Sahoo S. S. Valdez J. Kim M. Rueschman M. Redline S. (2019). ProvCaRe: Characterizing Scientific Reproducibility of Biomedical Research Studies using Semantic Provenance Metadata. *Int. J. Med. Inform.* 121 10–18. 30545485 10.1016/j.ijmedinf.2018.10.009 PMC6343667

[B39] Shen W. Wang J. Han J. (2015). Entity linking with a knowledge base: Issues, techniques, and solutions. *IEEE Trans. Knowl. Data Eng.* 27 443–460.

[B40] Sim I. Tu S. W. Carini S. Lehmann H. P. Pollock B. H. Peleg M. (2014). The Ontology of Clinical Research (OCRe): an informatics foundation for the science of clinical research. *J. Biomed. Inform.* 52 78–91. 10.1016/j.jbi.2013.11.002 24239612 PMC4019723

[B41] Soto A. J. Przybyla P. Ananiadou S. (2019). Thalia: Semantic search engine for biomedical abstracts. *Bioinformatics* 35 1799–1801. 10.1093/bioinformatics/bty871 30329013 PMC6513154

[B42] Tu S. W. Peleg M. Carini S. Bobak M. Ross J. Rubin D. (2011). A practical method for transforming free-text eligibility criteria into computable criteria. *J. Biomed. Inform.* 44 239–250. 20851207 10.1016/j.jbi.2010.09.007 PMC3129371

[B43] Turner J. A. Laird A. R. (2012). The cognitive paradigm ontology: Design and application. *Neuroinformatics* 10 57–66. 21643732 10.1007/s12021-011-9126-x PMC3682219

[B44] Turner M. D. Chakrabarti C. Jones T. B. Xu J. F. Fox P. T. Luger G. F. (2013). Automated annotation of functional imaging experiments via multi-label classification. *Front. Neurosci.* 7:240. 10.3389/fnins.2013.00240 24409112 PMC3864256

[B45] University of Bath (2023). *Finding and reusing research datasets: Finding Data Home.* Bath: University of Bath.

[B46] Viviano J. D. Buchanan R. W. Calarco N. Gold J. M. Foussias G. Bhagwat N. (2018). Initiative in neurobiology of the schizophrenia, resting-state connectivity biomarkers of cognitive performance and social function in individuals with schizophrenia spectrum disorder and healthy control subjects. *Biol. Psychiatry* 84 665–674. 10.1016/j.biopsych.2018.03.013 29779671 PMC6177285

[B47] Wallis J. C. Rolando E. Borgman C. L. (2013). If we share data, will anyone use them? Data sharing and reuse in the long tail of science and technology. *PLoS One* 8:e67332. 10.1371/journal.pone.0067332 23935830 PMC3720779

[B48] Walters W. H. (2020). Data journals: Incentivizing data access and documentation within the scholarly communication system. *Insights UKSG J.* 33:18.

[B49] Wang L. Alpert K. I. Calhoun V. D. Cobia D. J. Keator D. B. King M. D. (2016). SchizConnect: Mediating neuroimaging databases on schizophrenia and related disorders for large-scale integration. *Neuroimage* 124 1155–1167. 10.1016/j.neuroimage.2015.06.065 26142271 PMC4651768

[B50] Wang X. Wang Y. (2022). *Sentence-Level Resampling for Named Entity Recognition.* Seattle, US: Association for Computational Linguistics.

[B51] Wang X. Wang Y. Ambite J. L. Appaji A. Lander H. Moore S. M. (2022). Enabling Scientific Reproducibility through FAIR Data Management: An ontology-driven deep learning approach in the NeuroBridge Project. *AMIA Annu. Symposium Proc.* 2022 1135–1144. 37128458 PMC10148274

[B52] Widom J. (2008). “Trio: A System for Data, Uncertainty, and Lineage,” in *Managing and Mining Uncertain Data*, ed. Aggarwal C. (Berlin: Springer).

[B53] Wilkinson M. D. Dumontier M. Aalbersberg J. Appleton G. Axton M. Baak A. (2016). The FAIR Guiding Principles for scientific data management and stewardship. *Sci. Data* 3:160018. 10.1038/sdata.2016.18 PMC4792175 26978244

[B54] Wu H. Toti G. Morley K. I. Ibrahim Z. M. Folarin A. Jackson R. (2018). SemEHR: A general-purpose semantic search system to surface semantic data from clinical notes for tailored care, trial recruitment, and clinical research. *J. Am. Med. Inform. Assoc.* 25 530–537. 10.1093/jamia/ocx160 29361077 PMC6019046

